# Supporting a definition of predatory publishing

**DOI:** 10.1186/s12916-020-01599-6

**Published:** 2020-05-08

**Authors:** Edoardo Aromataris, Cindy Stern

**Affiliations:** grid.1010.0 0000 0004 1936 7304 JBI, Faculty of Health and Medical Sciences, The University of Adelaide, Adelaide, Australia

**Keywords:** Predatory publishing, Predatory journals, Publication ethics, Communication, Dissemination

## Background

Predatory publishing and the threat it poses to the scientific establishment and our dogmatic processes of dissemination, centred on publication and conference, have received increasing recognition over the last decade. Evolving publishing practices, including the shift from print to online publication and the increasing demand by authors and national funding agencies for open access models of publishing [1, 2], coupled with the long-standing academic mantra of ‘publish or perish’, have ensured a sustainable environment for an ever-increasing number of predatory journals to appear and also, unfortunately, flourish.

## Identifying predatory journals

Identifying a predatory journal is not always straightforward. With every continued success at attracting new manuscript submissions, whether unsuspecting or deliberate, predatory publishers have continued to evolve their undesirable art form into sophisticated operations that appear to be, at face value, legitimate. The systematic review presented by Cukier et al. [3] searched multiple and diverse publication sources from the last 8 years, including websites and YouTube as well as scientific journals, to identify and compare available checklists to help detect predatory journals. The methodological review identified 93 checklists, developed to help vigilant readers, authors and editors identify predatory journals. The sheer number identified underpins the cause for concern across academic disciplines posed by predatory publishing; however, as the author’s note [3] may well confound the author’s confidence in their utility—why are so many necessary?

On this point, the work presented by Cukier and colleagues [3] poses somewhat of a conundrum for authors; considering the number available, these authors caution against the development of further checklists but rather recommend authors to look for a checklist that provides a threshold value, or cut-off, and one that has been developed using rigorous evidence to assess predatory journals. Despite their comprehensive and rigorous review of those available, only one identified checklist fulfilled these criteria as recommended [3]. So where to then, from here? Until we see the demise of predatory publishing, the immediate goal should be for readers, authors, and editors to be able to easily identify a predatory journal. The work undertaken by Cukier et al. [3] plays an important role by providing us with a baseline of the current state of evidence within the biomedical field that will be expanded on over time.

## Supporting a definition

The available checklists offer a way forward. The review [3] also assessed the thematic content of the included checklists—where checklist items were directing the user towards. Despite their independent development, across a diverse range of publication sources and disciplines (including for example nursing, emergency medicine, psychiatry), the majority of checklists directed the user to the same details for assessment of the periodical; with over 75% of the checklists including questions related to journal business operations, editorial and peer review processes and practices, and/or the manner of communication between the journal/publisher and the authors, editors and readers. Less frequently, checklists directed users to an assessment of article processing charges, indexing, archiving and dissemination of content and finally towards an assessment of the quality of previously published articles.

As may be expected, the content of these checklists resonates with the general principles for identifying predatory, and on the other hand, legitimate, publishers; in short, similar cues appear repeatedly across checklists and other available reputable resources to help authors, including guidance by the Committee of Publishing Ethics (COPE) [4] and the Think.Check.Submit. campaign (https://thinkchecksubmit.org/). What would be an interesting exercise would be to compare the findings against those checklists that aim to identify ‘legitimate’ or ‘trustworthy’ journals to ascertain whether they are in fact parallel.

Indeed, the work by Cukier et al. [3] proved seminal in informing the recent definition for what the key characteristics of a predatory journal are and how to help authors identify them [5]. A consensus group of international experts recently agreed the key features of a predatory journal include presenting false or misleading information, non-adherence to best editorial and production practices, a lack of transparency and indiscriminate solicitation practices [5]. Interestingly, the consensus group pointedly and controversially omitted quality of peer review, a domain targeted by many of the identified checklists as well as the quality of the journal, on the basis that these were considered too subjective to include in any definition [3].

## Conclusions

In conclusion, we are left with ample cues and resources as authors to help us make informed decisions as to where we submit our work for publication. Ideally, a gold standard checklist will evolve, perhaps based on those identified by the review; however, further validation work is needed first, as well as a clearer way to assess the risk of bias between checklists.

## Data Availability

Not applicable

## Abbreviation

COPE: Committee of Publishing Ethics
